# vMUS-dBG: A Novel De Bruijn Graph Model for De Novo Genome Assembly Using Variable-Length Minimum Unique Substrings

**DOI:** 10.21203/rs.3.rs-8997819/v1

**Published:** 2026-04-27

**Authors:** Andrews Frimpong Adu, Elliot Sarpong Menkah, Peter Amoako-Yirenkyi, Samson Pandam Salifu

**Affiliations:** 1 Biochemistry and Biotechnology, Kwame Nkrumah University of Science and Technology, KNUST, Kumasi Ghana.; 2 Mathematics, Kwame Nkrumah University of Science and Technology, KNUST, Kumasi Ghana.; 3 Chemistry, Kwame Nkrumah University of Science and Technology, KNUST, Kumasi Ghana.

**Keywords:** minimum unique substring, repeats, suffix tree, sequence uniqueness, de bruijn graphs, ***v***MUS

## Abstract

De novo genome assembly using de Bruijn graphs (DBGs) typically relies on fixed-length k-mers as the nodes of the graph. While this approach is effective, it presents a fundamental trade-off: smaller k values tend to collapse repeats, whereas larger k values can result in fragmentation, particularly in low-coverage regions. Although multi-k and variable-order methods help mitigate these issues, they still rely on fixed-length topology or heuristic parameter selection. In this work, we introduce a de Bruijn graph constructed from Minimum Unique Substrings (MUSs), substrings that appear exactly once within the genome. This new graph is referred to as the variable-length MUS de Bruijn graph (vMUS-dBG). In the vMUS-dBG, the nodes are defined by read-extracted MUS anchors, and directed edges represent read-supported transitions between successive occurrences of MUSs. Each edge is also enhanced with instance-level metadata to preserve positional weights (repeats) and support counts. This innovative design eliminates the necessity for a global k-mer selection, while producing a concrete, repeat-aware graph construction that operates differently from the abstract manifold-style DBG model. Our experiments using real 24x *E. coli K12* HiFi data demonstrate that a prototype implementation of our approach achieves contiguity and accuracy comparable to that of a fixed-k method. These results establish MUS-based variable-length graph construction as a principled and biologically grounded alternative to fixed-k De Bruijn graph assembly to explore.

## 1 Introduction

Genome assembly algorithms have historically used graph-based models to represent and reconstruct DNA sequences from fragmented reads produced by sequencing technologies. Early methods based on overlap graphs [1], depict reads as nodes and represent the suffix-prefix overlaps as edges. This approach reduces assembly to the problem of finding a Hamiltonian path (path that visits every node exactly once), which is conceptually straightforward but leads to an NP-complete problem and does not scale well with modern high-throughput sequencing datasets. The introduction of the de Bruijn graph (DBG) model significantly transformed this field by rephrasing genome assembly as an Eulerian path problem [2–4]. In a DBG, input reads are first broken into substrings of fixed length k, called k-mers, which represent the graph nodes, and edges are represented by (k+1)-mers. The genome is then reconstructed from the Eulerian path in the graph. This redefinition enabled more efficient and scalable assembly methods and remains the basis for many contemporary assemblers.

Although highly effective and efficient, the de Bruijn strategy is limited by the requirement to use a global fixed value for k-mer length. This value is determined by the user and introduces a parameter bias that lacks a universally optimal solution. The choice of k therefore, has a significant impact on the quality of the assembly output [5]. Selecting smaller k values may improve coverage in low-depth regions but can lead to the collapse of repetitive sequences. This results in a tangled graph that limits the length of contiguous sequences (contigs). On the contrary, larger k values help resolve these repeats but are more susceptible to fragmentation. Even a slight drop in coverage can break the graph into disconnected components. Both scenarios lead to suboptimal and fragmented assemblies. This instability means that the graph?s topology can change significantly based on the user?s choice of k.

To address this trade-off, many modern assemblers employ multi-k and adaptive strategies, including iterative graph construction [6–8] and variable-order graphs [9–12]. The iterative approach increases the value of k in each iteration, while variable-order graphs represent all orders of k in a single data structure. Although these methods enhance robustness, they still rely on fixed-length k-mers as the primary graph components, necessitating heuristic decisions about parameter selection and graph simplification. Moreover, [9] introduced the manifold de Bruijn graph as an attempt to avoid the need to settle on a single k-mer length at any stage, but it has been of theoretical interest [8, 12]. These graphs generalize DBGs by allowing substrings of arbitrary length as nodes of the graph instead of fixed-length k-mers. Technically, these approaches tend to address the symptoms of the problem rather than its underlying structural limitations, resulting in poor performance with complex datasets, such as polyploid or cancer genomes [13].

In this study, we introduce the variable-length Minimum Unique Substring de Bruijn graph (vMUS-dBG). This is a practical assembly graph model where graph nodes consist of variable-length minimum unique substrings (MUSs) and edges represent read-supported transitions between successive MUS occurrences. A MUS is the shortest substring that uniquely identifies a region in the genome, with its length determined adaptively based on the local sequence context [14, 15]. This property is transformative because, by definition, a MUS must lie at the boundary between a repetitive region [14, 15]. Therefore, any graph constructed from MUSs will automatically have these repeat boundaries encoded in its structure, reducing redundancy and complexity of genome reconstruction.

This model, therefore, eliminates the necessity for a global k-mer value and makes the assembly process dependent solely on the inherent information present in the genome. The vMUS-dBG is motivated by [9] and this is the first implementation of such approach to our best of knowledge. We evaluate vMUS-dBG on a sampled real data, demonstrating the assembly reconstruction from MUSs. We also evaluate the graph construction and traversal processes, resource usage, and highlighting its scalability.

## 2 Conceptual and Theoretical Framework

### 2.1 Preliminaries

Since assembly tools employ varying terminology, we provide definitions tailored to the graph structures used in this work. All graphs considered are directed. Let G=(U,V) denote a graph with node set U and edge set V. A node u *precedes* (resp. *follows*) a node v if there is an edge u→v (resp. v→u) in G. The degree of u, denoted deg(u), is the number of edges incident to u. The in-degree and out-degree of u are ${\text{deg}}^{-}(u)$ and ${\text{deg}}^{+}(u)$, which represent the number of nodes preceding and following u, respectively. A node with ${\text{deg}}^{-}(u)=0$ is a *source*, whiles a node with ${\text{deg}}^{+}(u)=0$ is a *sink*. A node u is *1-in-1-out* if ${\text{deg}}^{-}(u)={\text{deg}}^{+}(u)=1$.

For graphs $G_{1}=\left(U_{1},V_{1}\right)$ and $G_{2}=\left(U_{2},V_{2}\right)$, their union is $G_{1}\cup G_{2}=(U_{1}\cup U_{2},V_{1}\cup V_{2})$. A *walk* is a sequence of alternating nodes and edges in which consecutive elements are incident. A *path* is a walk that does not revisit nodes, beginning and ending at nodes of degree one, with all internal nodes of degree two. A *cycle* is a closed walk in which every node has degree two.

A node u is a *bifurcation* if it has more than one incoming edge or more than one outgoing edge. A node is a *sentinel* if it corresponds to the first or last k-mer of any input read. A *junction* is any node that is a bifurcation, a sentinel, or both. A directed path in G is a *maximal non-branching path* (MNBP) if its endpoints are junctions and all internal nodes are 1-in-1-out.

### 2.2 The De Bruijn Graphs

Let R be a DNA read over the alphabet Σ={A,C,G,T} of length N, and let $ℛ=\left\{R_{1},\ldots ,R_{m}\right\}$ be a set of reads. For indices i≤j, denote the substring R[i:j], and call any length-p substring of a read a p-mer. A p-mer is a *prefix* of R if it equals R[1:p], and a *suffix* if it equals R[N-p+1:N].

#### 2.2.1 De Bruijn Graph for a String

For a fixed p, the order-p de Bruijn graph $G_{p}(ℛ)=(U,V)$ is constructed as follows. Let F be the multiset of all p-mers extracted from the reads.

- **Nodes** U: Each node is a (p-1)-mer that appears as the prefix or suffix of some p-mer in $F:U=\left\{d\in \Sigma^{p-1}|\exists f\in F:d=\text{prefix}(f,p-1)\vee d=\text{suffix}(f,p-1)\right\}$.
- **Edges** V: Each directed edge corresponds to a p-mer and links the overlapping (p-1)-mer prefix and suffix:V={(prefix(f,p-1),suffix(f,p-1))∣f∈F}. Thus, for $v_{1}=a_{1}\ldots a_{p-1}$ and $v_{2}=a_{2}\ldots a_{p}$, an edge $v_{1}\to v_{2}$ exists whenever their (p-2)-overlap matches.

#### 2.2.2 De Bruijn Graph for a Collection of Strings

For a set of reads ℛ and an integer p, the order-p de Bruijn graph is obtained by taking the union of the graphs constructed from each read: $G(ℛ,p)=G\left(r_{1},p\right)\cup G\left(r_{2},p\right)\cup \cdots \cup G\left(r_{m},p\right)$, where nodes with identical (p-1)-mer labels are merged. A walk (or path) t in G(ℛ,p) is said to *spell* a read r if t is exactly the path corresponding to G(r,p).

## 3 Methods

### 3.1 From k-mers to Minimum Uunique Substrings (MUS)

**Fig. 1:**
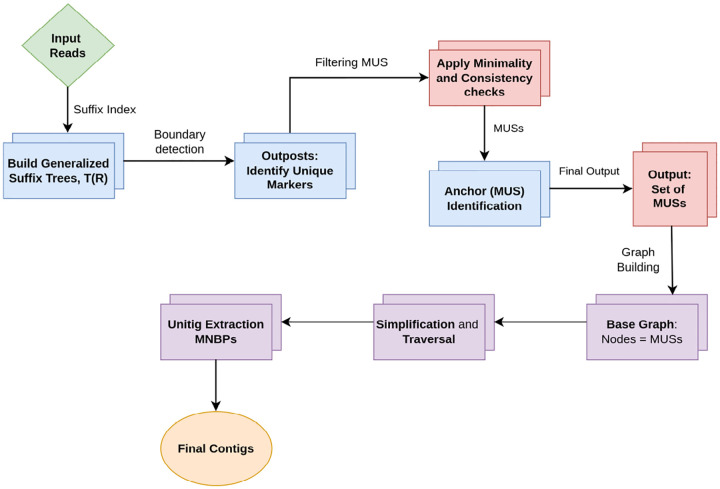
Variable-length MUS-dBG (vMUS-dBG) pipeline. Stage 1 (MUS Extraction): 1) The algorithm builds a generalized suffix tree 𝒯(ℛ) from the input read set ℛ. 2) It traverses this tree to identify outposts, which are unique prefixes of suffixes, and records their boundaries in the arrays ${\text{Right}}_{k}$ and ${\text{Left}}_{k}$. 3) It then determines the anchor set $A_{ℛ}(w)$, which serves as the operational definition of a minimal unique substring (MUS) for the reads. This procedure enforces three necessary properties–consistency, *left-minimality*, and *right-minimality*–to infer uniqueness in the set of reads. Stage 2 (Graph Construction): A MUS-based graph is then built from this set of MUSs, with nodes representing the MUSs and edges indicating the overlaps between consecutive MUSs. By traversing the MUS-based graph, unitigs are identified, leading to the construction and extraction of the final set of contigs.

Figure 1 illustrates the full vMUS-dBG construction pipeline. The process begins by extracting all minimum unique substrings (MUSs) from the read set. Let ℛ be the set of reads, and let $R_{k}\in ℛ$ be a read over the alphabet Σ. We denote by $\left|R_{k}\right|$ the length of $R_{k}$ and by $R_{k}[i:j]$ the substring spanning positions i to j. A substring w of $R_{k}$ is a MUS if it appears exactly once in the entire read set ℛ (considering both forward and reverse complements) whiles its proper prefixes or suffixes are repeats. A maximal repeat (MR), on the other hand, is a substring with an occurrence greater than 2 and cannot be extended on either side while still being a repeat [14]. The complete set of MUSs extracted from $R_{k}$ is denoted by ℳ.

To illustrate these definitions, consider the example read $R\left[\begin{array}{lll} 1 & : & 10 \end{array}\right]=\text{ATGCTAGCAC}$. A MUS is a substring that occurs exactly once in R and cannot be shortened at either end without losing its unique occurrence. In contrast, a repeat (maximal repeat) is a substring that occurs at least twice and cannot be extended to the left or right while preserving its repetitive nature. For this read, the MUSs are: {AT, TG, CT, TA, AG, GCA, AC }.

These MUSs coincide with the points where maximal repeats terminate, effectively acting as anchors at the boundaries of repeated regions. For example, the substring GC appears twice in R, at positions R[3:4] and R[7:8]. Each occurrence is immediately followed by a MUS: R[4:5]=CT and R[8:9]=CA. This illustrates the structural relationship between MUSs and MRs. Thus, MUSs mark the endpoints of maximal repeats and thus partition the read into uniquely anchored segments. For notational simplicity, we refer to all MUSs as *words*, denoted w(i,j)∈ℳ.

### 3.2 vMUS-dBG Representation

For the set of reads ℛ={ATGCTA,GCTAGC,TAGCAC,GCACAT,ACATGC} from the circular genome R, the extracted MUSs are $A_{ℛ}(w)=\{\text{AT},\;\text{TG},\;\text{CT},\;\text{TA},\;\text{AG},\;\text{GCA},\;\text{AC}\}$, as shown in Table 1.

We denote an occurrence of a MUS w∈ℳ in a read $R_{k}$ by (w,k,[s,e]), where k indicates which read, s is the start index and e is the end index of w in $R_{k}$. The assembly graph is a directed multigraph $G_{w}(ℛ)=(U,V)$ where:

- The set of nodes U is composed of the collection of all MUSs (𝒜) and two special terminal nodes (for when the MUS do not start or end the read): a source node, $U_{\text{start}}$, and a sink node, $U_{\text{end}}$ (Table 1). Formally, $U=A_{ℛ}(w)\cup \left\{U_{\text{start}},U_{\text{end}}\right\}$. Thus, $U=\left\{AT,TG,CT,TA,AG,GCA,AC,U_{\text{start}},U_{\text{end}}\right\}$.
- The edge set V represent the structural transitions and genomic intervals between them.

For each directed edge (u,v)∈V, we assign a multiplicity f(u,v) to represent the number of times the adjacency of that edge is observed across all reads. Multiple reads can induce the same directed edge pair (u→v). We formally define a flow function f:U×U→N as follows:

f(u,v)=the number of reads that produce the edge(u→v).

As a result, the edge set forms a multiset where each edge transition (u→v) has a multiplicity of f(u,v)≥1. Additionally, for every edge instance (u,v), we maintain a list of attributes. For edges that correspond to two consecutive MUSs within a read, these attributes are:

- **Weight** (δ): Each occurrence of (u→v) in a read is annotated with a weight value describing the positional offset between the MUSs. If the MUS at node u occurs at positions $\left[s_{u},e_{u}\right]$ and the MUS at node v at $\left[s_{v},e_{v}\right]$ in $R_{k}$, then the weight is
(1) $$\delta (u,v)=s_{v}-s_{u}.$$
- **Weight Tag** (τ): The explicit sequence representing the repeat unit between two MUSs:
(2) $$\tau =\left\{\begin{array}{ll} R_{k}\left[e_{u}+1:s_{v}-1\right], & \delta >|u|,\\ \varepsilon , & (\text{overlap case/adjacent}), \end{array}\right.$$
  where ε is the empty string.

#### 3.2.1 Edge Construction Rules

For each edge occurrence contributed by a read $R_{k}$, we record the paired label (δ,τ). Thus, every directed edge u→v accumulates a list of such labels, denoted {(δ,τ)}, aggregated over all reads in which the edge occurs. These labels encode both the relative offset between consecutive MUSs. We define two types of edge annotations: (1) unique edges, where the consecutive MUSs are adjacent (overlap) (2) repeat edges, where there is a gap and a MR covers the intervals. We denote all such collection of labels for an edge type (u,v) by ℐ(u,v). To construct these edge labels, we distinguish three cases:

- **Prefix Edges (Initial Substring).** If the first MUS $\left(w_{1},\left[s_{1},e_{1}\right]\right)$ occurrence begins at position $s_{1}>1$, then it does not start at the beginning of $R_{k}$. In this case, we set $\delta =s_{1}-1$ and define the tag as the prefix
$$\tau =R_{k}[1:\delta ],$$
  corresponding to the initial segment of the read preceding the first MUS.
- **Internal Edges (Consecutive MUSs).** For each consecutive pair of MUSs $\left(w_{i},\left[s_{i},e_{i}\right]\right)$ and $\left(w_{i+1},\left[s_{i+1},e_{i+1}\right]\right)$ in $R_{k}(\text{for}\;1\leq i\leq p-1)$, we set
$$\delta =s_{i+1}-s_{i}.$$
  If δ>|u|, then v begins after u ends, indicating a gap (as illustrated in Equation 2). We therefore assign
$$\tau =R_{k}\left[\left(e_{i}+1\right):\left(s_{i+1}-1\right)\right],$$
  the intervening substring between the two MUSs. Otherwise, when δ≤|u|, the MUSs overlap, and we set τ=ε (the empty string).
- **Suffix Edges (Final Substring).** If the last MUS ends at position $e_{p}<\left|R_{k}\right|$, then the read contains a trailing segment not covered by any MUS. We record
$$\delta =\left|R_{k}\right|-e_{p},\;\tau =R_{k}\left[\left(e_{p}+1\right):\left|R_{k}\right|\right],$$
  the suffix of the read following the final MUS.

#### 3.2.2 Node Properties (Prefix/Suffix Maximization)

For each node u∈U, we maintain auxiliary metadata that summarizes the appearance of u across the read set. Let P(u) represent the property set associated with u, which includes two key descriptors:

- **Maximal Prefix:** The longest sequence of characters that precedes any occurrence of u in the reads, together with its length.
- **Maximal Suffix:** The longest sequence of characters that follows any occurrence of u in the reads, together with its length.

**Fig. 2:**
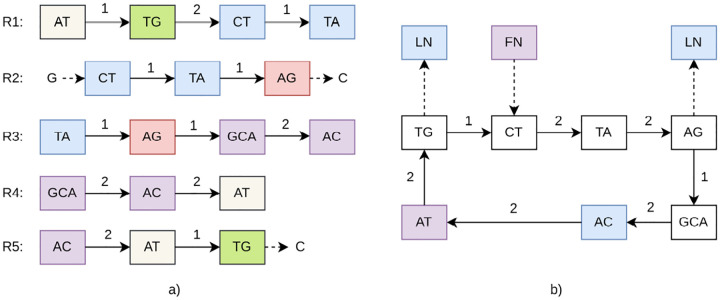
vMUS-dBG representation for a set of reads ℛ={ATGCTA,GCTAGC,TAGCAC,GCACAT,ACATGC} from the circular genome R=ATGCTAGCAC. (a) All MUSs $A_{ℛ}(w)=\{\text{AT},\;\text{TG},\;\text{CT},\;\text{TA},\;\text{AG},\;\text{GCA},\;\text{AC}\}$ and the corresponding paths in ℛ annotated with shifts, $U_{\text{start}}$, and $U_{\text{end}}$. (b) The resulting $v\text{MUS-dBG}\left(ℛ,A_{ℛ}(w)\right)$.

In addition, we record analogous global descriptors: the maximal prefix over the entire read set at the special start node $U_{\text{start}}$, and the maximal suffix over the entire read set at the terminal node $U_{\text{end}}$. Collectively, these metadata and edge construction rules define the structure illustrated in Figure 2.

#### 3.2.3 The Assembly Graph

**Fig. 3:**
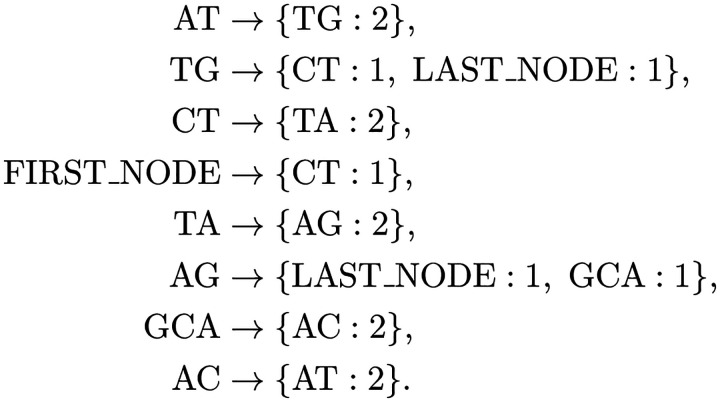
This is the assembly graph represented by using an adjacency list, where $U_{\text{start}}=\text{FIRST}\_\text{NODE}$ and $U_{\text{end}}=LAST_{-}\text{NODE}$. Each node corresponds to a MUS, in addition to *FIRST_NODE* and *LAST_NODE*. For Example, Node AT has outgoing neighbors TG, with a flow of 2. Node TG connects to outgoing neighbors CT and *LAST_NODE*. Node CT has outgoing neighbors leading to TA, and so on.

**Fig. 4:**
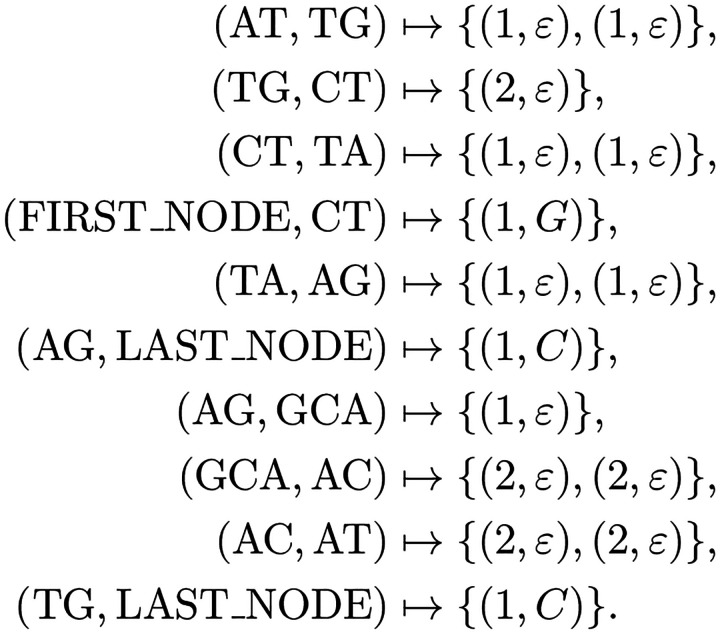
This figure depicts the edge data representation of the assembly graph. This includes tuples of the form (δ,τ), where each tuple encodes the weight length and the tag substring. The ε indicate an empty string indicating an overlap between consecutive MUSs. The flow function is defined as follows: C(AT,TG)=2,C(CT,TA)=2,C(AC,AT)=2, and so on. The annotation function is represented as: ℐ(AT,TG)={(1,ε),(1,ε)} and ℐ(TG,CT)={(2,ε)}, among other possible pairs.

**Definition 1** (Assembly Graph) The **assembly graph** is therefore given as a directed multigraph

$$G_{ℳ}=\left(U_{ℳ},V_{ℳ},f_{ℳ},{ℐ}_{ℳ},P{\left(u\right)}_{ℳ}\right),$$

where:

- $U_{ℳ}=\underset{i}{⋃}{ℳ}_{i}\cup \left\{U_{\text{start}},U_{\text{end}}\right\}$,
- $V_{ℳ}\subseteq U\times U$ is the multiset of directed edges,
- $f_{ℳ}:V\to N$ flow function or multiplicity, counting the number of times the adjacency (u,v) is observed,
- ${ℐ}_{ℳ}:V\to N\times \Sigma^{*}$ annotation function, and P(u) assigns to each vertex its prefix and suffix metadata. This is depicted in Figures Figure 2, 3, and 4.

The detail structure of the graph construction is described in the next section.

### 3.3 vMUS-dBG Construction

The assembly graph $G_{ℳ}=\left(U_{ℳ},V_{ℳ},f_{ℳ},{ℐ}_{ℳ},P(u{)}_{ℳ}\right)$ is now constructed by processing each read ${\text{read}}_{k}$ and its corresponding MUS occurrences. The construction of the graph is accomplished by iterating through each input read $R_{k}\in ℛ$. For every read, all occurrences of the MUS from the set $A_{R}(w)$ are identified. These occurrences are then sorted by their starting index to create an ordered sequence, $S_{k}$. The graph is generated by establishing directed edges between consecutive MUSs in this sequence, along with edges connecting the source node to the first MUS and from the last MUS to the sink node.

#### 3.3.1 vMUS-dBG Construction Rules:

The count function for the edge multiplicities (flows), $f_{ℳ}$, and the collection of edge data lists, ${ℐ}_{ℳ}$, are initialized to zero and empty, respectively, for all possible node pairs (u,v). Similarly, the data structure P(u) is initialized for all nodes u to track maximal prefix and suffix information.

- **Prefix Segment:** For a given read $R_{k}$, if its first identified MUS, $w_{1}$, starts at index $s_{1}>1$, a directed edge is established from the source node, $U_{\text{start}}$, to $w_{1}$. The flow of this edge, $f\left(U_{\text{start}},w_{1}\right)$, is incremented. The edge data for this instance is defined by a weight $\delta =s_{1}-1$ and a tag $\tau =R_{k}\left[1:s_{1}-1\right]$, and these are appended to $ℐ\left(U_{\text{start}},w_{1}\right)$. Additionally, the maximal prefix information in $P\left(w_{1}\right)$ and $P\left(U_{\text{start}}\right)$ is updated if the current prefix length is greater than the previously recorded length.
- **Internal Segments:** For each pair of consecutive MUS, $\left(w_{i},\left[s_{i},e_{i}\right]\right)$ and $\left(w_{i+1},\left[s_{i+1},e_{i+1}\right]\right)$, in the sorted sequence of MUS for a read, a directed edge is established from $w_{i}$ to $w_{i+1}$. The flow $f\left(w_{i},w_{i+1}\right)$ is incremented. The edge data for this instance is defined by a weight $\delta =s_{i+1}-s_{i}$ and a tag $\tau =R_{k}\left[e_{i}+1:s_{i+1}-1\right]$, which are appended to $ℐ\left(w_{i},w_{i+1}\right)$.
- **Suffix Segment:** For a given read $R_{k}$, if its last identified MUS, $w_{p}$, ends at index $e_{p}<\left|R_{k}\right|$, a directed edge is established from $w_{p}$ to the sink node, $U_{\text{end}}$. The flow $f\left(w_{p},U_{\text{end}}\right)$ is incremented. The edge data for this instance is defined by a weight $\delta =\left|R_{k}\right|-e_{p}$ and a tag $\tau =R_{k}$, which is appended to $ℐ\left(w_{p},U_{\text{end}}\right)$. The maximal suffix information in $P\left(w_{p}\right)$ and $P\left(U_{\text{end}}\right)$ is updated if the current suffix length is greater than the previously recorded length.

The algorithm returns the populated graph represented by the tuple (f,ℐ,P), where f is the count function for edge multiplicities, ℐ is the collection of edge data, and P is the terminal data for each node, which fully describes the constructed assembly graph. This is depicted in Algorithm 1 and Figure 2 (a) and (b).

## 4 Graph Simplification and Traversal

### 4.0.1 Degree Calculation and Branching Logic

A key innovation of this algorithm is its redefinition of what constitutes a “branching/junction” point within a graph. Specifically, the definition of a branching node (junction) is determined by the count of distinct edges, considering both the total number of incoming and outgoing connections. In other words, if ${\text{deg}}^{+}(u)>1$, there are multiple ways to depart from the node u, even if all paths lead to the same node v. Therefore, a choice must be made regarding which specific edge instance to traverse, and this choice point is what constitutes a branch. Given the directed multigraph model $G_{ℳ}=(U,V,f,ℐ,P(u))$, the set of edge instances associated with an edge (u,v) is defined as:

$$ℐ(u,v)={\left\{\left(\delta_{i},\tau_{i}\right)\right\}}_{i=1}^{m},$$

where each pair $\delta_{i}\in Z_{\geq 0}$ is the weight and $\tau_{i}\in \Sigma^{*}$ is the tag as defined in Equation 3.2. We therefore, distinguishes between two types of graph degrees:

- Total Degrees (Flow): This represents the sum of all edge instances entering or leaving a node, including parallel edges. It serves as a measure of overall read support.
- Unique Degrees: This counts the number of distinct nodes connected to a given node, disregarding multiple parallel edges between the same two nodes and instead focusing on the unique paths available.

Formally, for any node u∈U, the total in-degree is given as $f^{-}(v)=\Sigma_{v\in U}f(u,v)$ and the total out-degree is $f^{+}(v)=\Sigma_{w\in U}f(v,w)$. Moreover, for any node u∈U, the unique in-degree is ${\text{deg}}^{-}(v)=|\{v\in U|f(u,v)>0\}|$ counting distinct predecessors, and the unique out-degree ${\text{deg}}^{+}(v)=|\{w\in U|f(v,w)>0\}|$ counting distinct successors. These considerations lead to the following definition:

**Definition 2** (Junction Node) A node u∈U is a junction if the following holds: $u\in \left\{U_{\text{start}},U_{\text{end}}\right\},{\text{deg}}^{-}(u)\neq 1$, or ${\text{deg}}^{+}(u)\neq 1$.


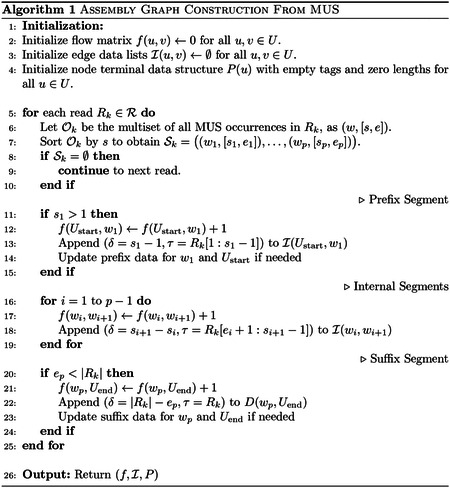


**Definition 3** (Source/Sink:) A node u∈U is source node if ${\text{deg}}^{-}(u)=0$ and ${\text{deg}}^{+}(u)>0$. A node v∈ℳ is sink node if ${\text{deg}}^{+}(v)=0$ and ${\text{deg}}^{-}(v)>0$.

**Proposition 1** (Best Edge Instance Selection:) *Given an edge* (u,v) *and a list of its associated data tuples* ℐ(u,v), *the best instance* (δ,τ) *is selected based on the following rules*:

- *Terminal Edges*: *If* $u=U_{\text{start}}$ *or* $v=U_{\text{end}}$, *the best instance is the one with the maximum length of* τ. *In case of a tie, the instance with the smallest weight* δ *is chosen*.
- *Internal Edges: For all other edges, the best instance is the one with the smallest weight* δ. *In case of a tie, the instance with the maximum length of* τ *is chosen*.

This is given mathematically as:

$$\left(\delta^{*},\tau^{*}\right)=\left\{\begin{array}{ll} \text{arg}\underset{\left(\delta ,\tau )\in 𝒯(u,v\right)}{\text{max}}(|\tau |,-\delta ), & \text{if}\;u=U_{\text{start}}\;\text{or}\;u=U_{\text{end,}}\\ \text{arg}\underset{\left(\delta ,\tau )\in 𝒯(u,v\right)}{\text{min}}(\delta ,-|\tau |), & \text{otherwise}. \end{array}\right.$$

### 4.1 Path Extension and Sequence Construction

#### 4.1.1 Graph Simplification and Unitig Extraction

After the assembly graph construction, we simplify $G_{ℳ}$ by removing trivial linear paths. We call a node $u\in U\setminus \left\{U_{\text{start}},U_{\text{end}}\right\}$ strictly linear if it has exactly one unique predecessor and successor, and the total flow of 1. That is, ${\text{deg}}^{-}(v)=1\wedge {\text{deg}}^{+}(v)=1\wedge f^{-}(v)=1\wedge f^{+}(v)$.

We now describe the path collapsing operation. Let P=(u,v,w) be a path of nodes in the $G_{ℳ}$ where v is a strictly linear node. Let $e_{1}=(u,v)$ be the unique incoming edge and $e_{2}=(v,w)$ be the unique outgoing edge. Let $ℐ\left(e_{1}\right)=\left(\delta_{1},\tau_{1}\right)$ and $ℐ\left(e_{2}\right)=\left(\delta_{2},\tau_{2}\right)$, then the path P is collapsed by modifying $G_{ℳ}$. This is achieved by removing $v,e_{1}$, and $e_{2}$ from the path and adding a new edge $e_{12}=(u,w)$. The new edge now has $f\left(e_{12}\right)=1$ and $ℐ\left(e_{12}\right)=\left(\delta_{1}+\delta_{2},\tau_{1}⊕v⊕\tau_{2}\right)$, where ⊕ denotes string concatenation. From this, we now obtain a simplified graph given as $G_{S}=(U,V,f,ℐ)$ after an iterative application of the graph collapsing operation until no strictly linear nodes remain.

#### 4.1.2 Unitig Extraction

Unitigs are then extracted from $G_{S}$ by decomposing it into a set of maximal non-branching paths. Thus, in $G_{S}$, a node v∈U is a junction if ${\text{deg}}^{-}(v)\neq 1$, or ${\text{deg}}^{+}(v)\neq 1$.

**Definition 4** (Maximal Non-Branching Paths, MNBP) A MNBP is a path $P=\left(v_{0},v_{1},\ldots ,v_{k}\right)$ in $G_{𝒮}$ such that (1) $v_{0}$ is a source node, or junction, (2) $v_{k}$ is a sink, or junction, (3) All internal nodes $v_{1},\ldots ,v_{k-1}$ are non-branching, and (4) Each edge instance (δ,τ)∈ℐ(u,v) is used at most once across all extracted paths. Unitigs correspond to MNBP in the assembly graph.

The sequence S(P) is now constructed by concatenating the sequence of each node with the sequence tag from the preceding edge in $G_{S}$. The reconstruction is built as follows given the path $P=\left(v_{0},v_{1},\ldots ,v_{k}\right)$ (Algorithm 2). Let seq(v) denote the nucleotide sequence of node v, and let the edge $e_{i}=\left(v_{i},v_{i+1}\right)$ be associated with a distance $\delta_{i}$ and a weight tag $\tau_{i}$. The reconstructed sequence S(P) is defined as follows:

##### Initial Sequence (Base Case)

The starting sequence $S_{0}$ depends on the nature of the source node $v_{0}$:

(3) $$S_{0}=\left\{\begin{array}{ll} \tau_{0}\cdot \text{seq}\left(v_{1}\right) & \text{if}\;v_{0}=U_{\text{start}}\\ \text{seq}\left(v_{0}\right) & \text{if}\;v_{0}\neq U_{\text{start}} \end{array}\right.$$

where • denotes the string concatenation operator.

##### Recursive Path Extension

For each subsequent edge $\left(v_{i},v_{i+1}\right)$ where $v_{i+1}\neq U_{\text{end}}$, we define the overlap $\phi_{i}$ as:

(4) $$\phi_{i}=\left|\text{seq}\left(v_{i}\right)\right|-\delta_{i}$$

The sequence is extended iteratively such that $S_{i+1}=S_{i}\cdot \Delta \left(v_{i},v_{i+1}\right)$, where the transition component $\Delta \left(v_{i},v_{i+1}\right)$ is determined by the spatial relationship between the MUS anchors. Let $L_{i}=\left|\text{seq}\left(v_{i+1}\right)\right|-\phi_{i}$ be the length of the non-overlapping suffix. The transition component is defined as:

(5) $$\Delta \left(v_{i},v_{i+1}\right)=\left\{\begin{array}{ll} \text{suffix}\left(\text{seq}\left(v_{i+1}\right),L_{i}\right) & \text{if}\;\phi_{i}>0\\ \text{seq}\left(v_{i+1}\right) & \text{if}\;\phi_{i}=0\\ \tau_{i}\cdot \text{seq}\left(v_{i+1}\right) & \text{if}\;\phi_{i}<0 \end{array}\right.$$

##### General Form

For a path where $v_{0}\neq U_{\text{start}}$ and $v_{k}\neq U_{\text{end}}$, the complete genomic sequence is represented by the concatenation of the initial node and all subsequent transition components:

(6) $$S(P)=\text{seq}\left(v_{0}\right)\cdot \prod_{i=0}^{k-1}\Delta \left(v_{i},v_{i+1}\right)$$

**Definition 5** (Final Contig Assembly (Algorithm 2)) For each path $P_{U}=\left(U_{1},\ldots ,U_{k}\right)\in P_{\text{final}}$, reconstruct the base path $P_{B}$ by concatenating internal paths of the original unitigs. Then, apply the sequence reconstruction function using original edge labels $\lambda_{B}$ to generate the final contiguous sequence.


$$S\left(P_{B},\lambda_{B}\right)$$

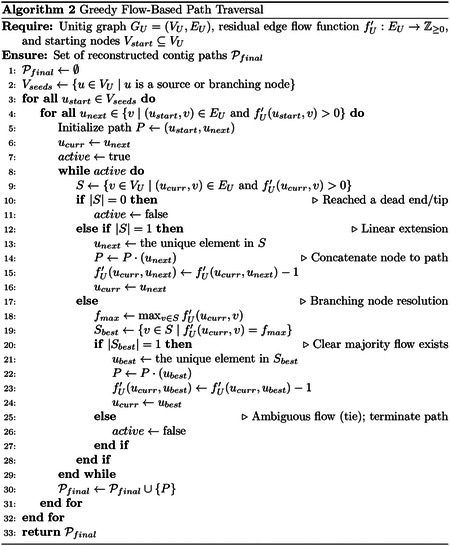


## 5 Experiments and Results

We built a de Bruijn graph using variable-length Minimum Unique Substrings (vMUS-dBG). The analysis was performed on a PowerEdge M620 (VRTX) server equipped with 640.0 GB of RAM and powered by two Intel(R) Xeon(R) CPU E5–2670 v2 processors running at 2.50 GHz, and running Ubuntu Server 22.04.5 LTS. The implementation is written in both C++ and Python, leveraging the advantages of each language. The source code for vMUS-dBG can be accessed at the provided link git@github.com:fandrew19/vMUS-dBG-Assembler.git.

**Fig. 6:**
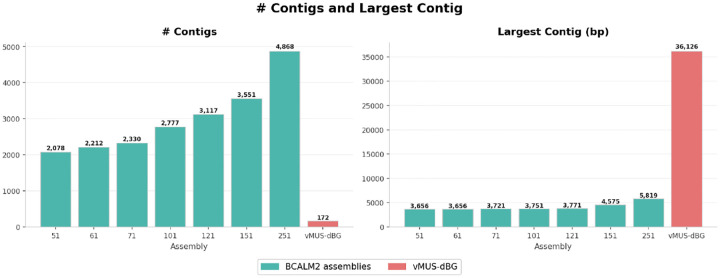
Assembly contig counts and the largest contiguous sequence observed across the fixed-k(k=51-251bp) approach and vMUS-dBG.

**Fig. 7:**
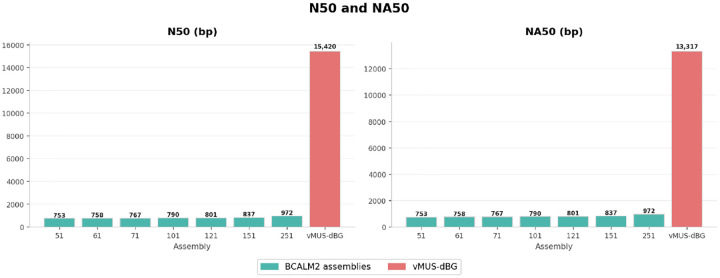
Assembly contiguity regarding N50 (raw contig lengths) and NA50 (aligned blocks) was higher for the vMUS-dBG method compared to the fixed-k approach at all k-values.

**Fig. 8:**
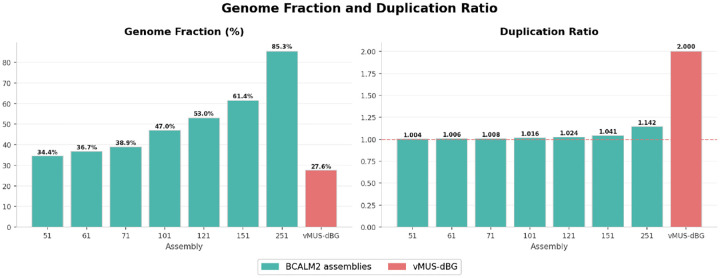
In terms of assembly completeness, the fixed-k approach outperforms vMUS-dBG, while vMUS-dBG shows a higher duplication ratio of 2.0.

**Fig. 9:**
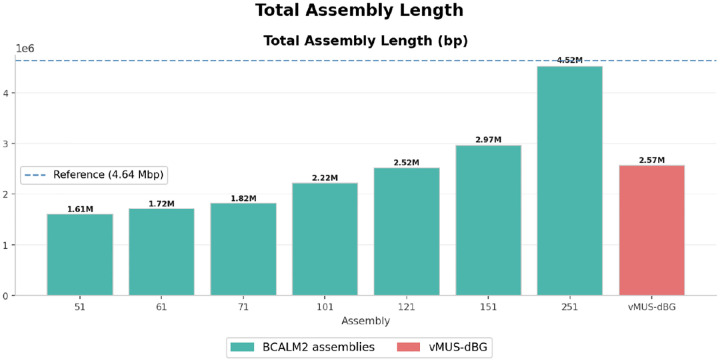
Total assembled sequences. Total assembled sequences showed that each k had different assembled lengths, ranging from 1.6 Mb to 4.5 Mb, compared to the vMUS-dBG, which measured 2.5 Mb.

We compared vMUS-dBG with BCalm2 [16] for graph construction with the default configuration DKSIZE_LIST=“326496128160192224256 320”. BCalm2 is a highly parallel implementation of an algorithm that first counts all the k-mers, then analyzes the overlaps between these k-mers to construct the Compressed De Bruijn Graph (CdBG). To ensure a fair comparison, the program is executed on a single core and with default parameters. We used HiFi data from *E. coli bacteria K12* with run identification SRR10971019. We randomly downsampled the reads to 24x coverage. We arbitrarily varied k-mer sizes (k values from 21 to 251) in BCalm2 to examine how the graph changes with varying k-mer sizes. We evaluated the assembly outputs from each run using QUAST [17]. Figures 6, 7, 8, and 9 depict the structural perturbations with respect to varying k, compared to our variable length construction.

Computationally, the fixed-k approach is more efficient than vMUS-dBG, using less memory and processing time across all k values. This efficiency stems from our custom implementation of the suffix tree which required significant amount of memory. Structurally, vMUS-dBG generates significantly fewer contigs (172), approximately 12 to 28 times fewer than any fixed-k assembly (2078–4868). However, it produces larger contigs (36,126 bp), which significantly outperform the 5,819 bp seen in the fixed-k-mer values as illustrated in Figure 6.

In terms of contiguity, we observe consistently short contigs across all k values, with N50 values ranging from 753 bp (k=51) to 972 bp (k=251). In contrast, the N50 value for vMUS-dBG is notably high at 15,420 bp, demonstrating adaptability approximately 16–20 times better than that of any fixed-k assembly. Furthermore, vMUS-dBG achieves a higher NA50 (13,317 bp) than the fixed k-mer approach across all tested k values (753bp-972bp). This is illustrated in Figure 7.

The genome fraction, which measures the extent of coverage of the reference genome, ranges from 34% (k=51) to 85% (k=251), with coverage steadily increasing as k increases. Nevertheless, none of the fixed-k assemblies cover the entire genome; even at k=251, there remains 15% uncovered, primarily due to graph fragmentation at higher k values. On the other hand, vMUS-dBG provides only 27.6% coverage, which is lower than that of k=51.

Additionally, the duplication ratio presents a complementary perspective. All fixed k assemblies maintain a duplication ratio close to 1.0 (ranging from 1.004 to 1.142), indicating virtually no redundancy. The slight increase at k=251 (1.142) suggests that the assembler begins to produce overlapping contigs as the graph structure becomes more complex at higher k values. In contrast, vMUS-dBG has a duplication ratio of 2.0, meaning that, on average, each assembled base is represented twice. This aligns with the approach’s unique-repeat awareness (Figure 8).

The total assembled length, which includes the reference length of 4, 641, 652 bp for context, reveals a key trade-off in assembly philosophy. The fixed-k assemblies range from 1.6 Mbp (for k=51) to 4.5 Mbp (for k=251), with k=251 coming closest to the reference length. However, even at k=251, the assembled bases comprise numerous short, fragmented contigs with minimal errors. In contrast, vMUS-dBG assembles 2.57 Mbp, falling between k=101 and k=121 in raw length, but it delivers much better contiguity and contig quality (Figure 9).

### 5.1 Discussion

This analysis highlights a fundamental trade-off between the breadth of coverage and the continuity of assembly. The fixed k-mer approach is effective at capturing a large portion of the genome but struggle with resolving repeats, leading to a fragmented assembly consisting of very short contigs. In contrast, the vMUS-dBG method effectively leverages variable-length information to overcome the ambiguities that fixed-k methods face. By employing minimum unique substrings instead of fixed k-mers, vMUS-dBG produces contigs that are significantly longer. However, the high duplication ratio (2.0) and discrepancy between N50 and NA50 indicate that vMUS-dBG preserves repeat ambiguity as alternative paths rather than collapsing them. This is by design; the graph encodes uncertainty rather than forcing incorrect resolution.

### 5.2 Conclusion

We introduced a new method, the variable-length Minimum Unique Substring-based de Bruijn graph (vMUS-dBG), that integrates sequence uniqueness and repeat boundaries directly into the graph’s structure. By embedding these characteristics into the topology of the graph, the vMUS-dBG reconceptualizes repeat resolution as an inherent property of the graph, rather than a parameter-sensitive outcome that depends on a fixed k-mer choice.

Evaluation using HiFi sequencing data from E. coli K-12 demonstrates that the vMUS-dBG effectively prevents catastrophic repeat collapse and excessive fragmentation often observed in fixed-k graphs. These findings establish Minimum Unique Substrings as both a biologically relevant and algorithmically feasible basis for repeat-aware genome assembly.

The method is expected to benefit significantly from higher sequencing depth, as increased coverage will produce more overlaps between MUS and enhance graph connectivity. Additionally, it will improve with rigorous error correction since single-base errors can disrupt uniqueness and lead to false MUS outcomes. In this study, our scalability was limited by the memory requirements of constructing generalized suffix trees and extracting MUS in our custom implementation, which restricted experimentation with higher-coverage datasets on standard hardware.

Switching from a suffix tree to a suffix-array-based framework substantially reduces memory overhead and enables practical deployment on typical computing systems, thereby enhancing scalability without compromising the method’s theoretical foundation.

## Figures and Tables

**Fig. 5:**
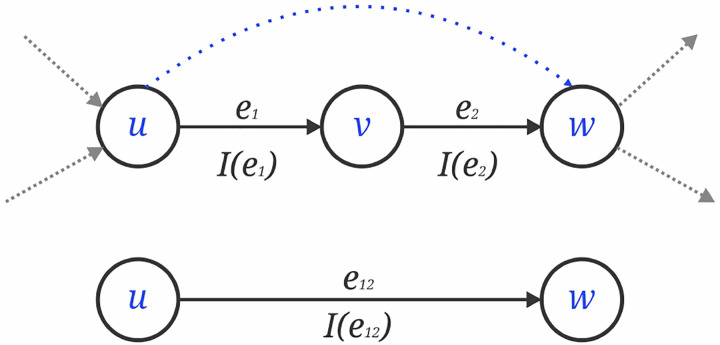
Graph simplification where node v and edges $e_{1},e_{2}$ removed and replaced with a new edge $e_{12}=(u,w)$.

**Table 1:** The set of MUSs extracted using Figure 1 for the set of reads ℛ from the genome R.

Read, $R_{k}$	MUS per read
ATGCTA	AT, TG, CT, TA
GCTAGC	CT, TA, AG
TAGCAC	TA, AG, GCA, AC
GCACAT	GCA, AC, AT
ACATGC	AC, AT, TG
